# Supplementary material for: TET1 dioxygenase is required for FOXA2-associated chromatin remodeling in pancreatic beta-cell differentiation
Source: Nat Commun. 2022 Jul 7;13:3907. doi: 10.1038/s41467-022-31611-x (PMC9263144; doi:10.1038/s41467-022-31611-x)
Supplement: Supplementary file 1 — Supplementary Information [file 41467_2022_31611_MOESM1_ESM.docx]

**­­Title: TET1 dioxygenase is required for FOXA2-associated chromatin remodeling in pancreatic beta-cell differentiation**

**Authors:** Jianfang Li^1,2,3#^, Xinwei Wu^1#†^, Jie Ke^1#^, Minjung Lee^4^, Qingping Lan^1^, Jia Li^4‡^, Jianxiu Yu^5^, Yun Huang^4^, De-Qiang Sun^2,6*^, Ruiyu Xie^1,7*^

**Affiliations:**

^1^ Department of Biomedical Sciences, Faculty of Health Sciences, University of Macau, Macau SAR, 999078, China

^2^ Innovation Center for Advanced Interdisciplinary Medicine, the Fifth Affiliated Hospital of Guangzhou Medical University, Guangzhou, 510530, China.

^3^ Guangzhou Laboratory, Guangzhou 510005, China

^4^ Center for Epigenetics & Disease Prevention, Institute of Biosciences and Technology, College of Medicine, Texas A&M University, Houston, TX 77030, USA.

^5^ Department of Biochemistry and Molecular Cell Biology & Shanghai Key Laboratory of Tumor Microenvironment and Inflammation, Shanghai Jiao Tong University School of Medicine, Shanghai 200025, China

^6^ Cardiology Department, the Second Affiliated Hospital, Zhejiang University School of Medicine, Hangzhou 310009, China

^7^ Ministry of Education Frontiers Science Center for Precision Oncology, University of Macau, Macau SAR, 999078, China

# These authors contributed equally to the work.

† Current address: Thoracic Epigenetics Section, Thoracic Surgery Branch, Center for Cancer Research, National Cancer Institute, National Institutes of Health, Bethesda, MD 20892, USA.

‡ Current address: State Key Laboratory of Respiratory Disease, National Clinical Research Center for Respiratory Disease, Guangzhou Institute of Respiratory Health, the First Affiliated Hospital of Guangzhou Medical University, Guangzhou 510120, China

* Correspondence: ruiyuxie@um.edu.mo; deqiangs@zju.edu.cn

**
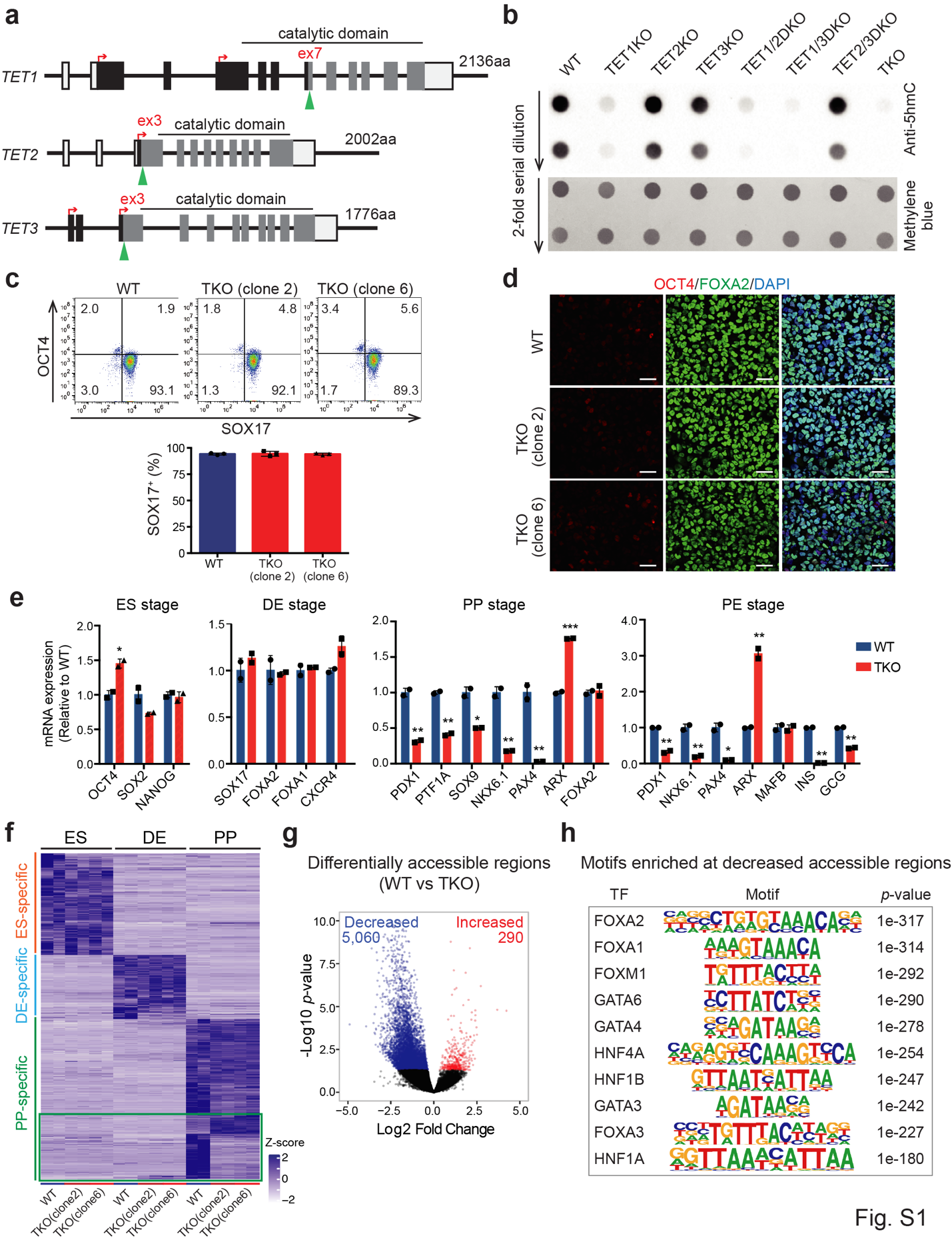
**

**Supplementary Fig. 1 Generation and characterization of TET-deficient cell lines.**

**a** TET-knockout mutants were generated using CRISPR gRNAs (green arrowheads) targeting exon 7 of *TET1,* exon 3 of *TET2*, and exon 3 of *TET3*. Translation start sites are indicated by red arrows. **b** Analysis of global 5-hydroxymethylcytosine (5hmC) levels (top) in WT, *TET1* knockout (TET1KO), *TET2* knockout (TET2KO), *TET3* knockout (TET3KO), *TET1/TET2* double knockout (TET1/2DKO), *TET1/TET3* double knockout (TET1/3DKO), *TET2/TET3* double knockout (TET2/3DKO), and *TET1/TET2/TET3* triple knockout (TKO) hESCs by 5hmC dot blot analysis. The bottom panel shows methylene blue staining using the total amount of input DNA as the loading control. **c** Representative plots of flow cytometry for the expression of pluripotency marker (OCT4) and endoderm marker (SOX17) at the DE stage are shown in the top panel. Quantification of the percentage of SOX17^+^ cells is shown in the bottom panel (n = 3 independent differentiation). Bar graph shows mean ± SD. **d** Immunostaining of OCT4 and FOXA2 in WT and TKO cells at the DE stage (n = 3 independent differentiations; scale bar = 50 μm). **e** Expression analysis by RT-qPCR for specific genes in WT and TKO (clone 2) cells at the ES, DE, PP, and PE stages. RT-qPCR validation was performed with three independent batches of samples (*p* = 0.0206 for *OCT4* in ES; *p* = 0.0050, *p* = 0.0024, *p* = 0.0132, *p* = 0.0052, *p* = 0.0094, and *p* = 0.0010 for *PDX1*, PTF1A, *SOX9*, *NKX6.1*, *PAX4*, and *ARX* in PP, respectively; *p* = 0.0048, *p* = 0.0099, *p* = 0.0103, *p* = 0.0049, *p* = 0.0015, and *p* = 0.0049 for *PDX1*, *NKX6.1*, *PAX4*, *ARX*, *INS*, and *GCG* in PE, respectively; student’s *t*-test, 2-sided; without multiple test correction). All bar graphs show mean ± SD. **f** Heatmap showing stage-specific DEGs in TKO compared with WT cells at the ES, DE, and PP stages (|fold change| ≥ 2; FDR < 0.05). Two mutant lines were used for TKO (clones 2 and 6). Each column represents one biological replicate for each cell line. Genes located in green rectangle are DEGs in TKO_PP compared with WT_PP cells. **g** Volcano plot of ATAC-seq data illustrating differentially accessible regions in TKO_PP cells (FDR < 0.05). **h** Transcription factor (TF) motif enrichment analysis of genomic regions showed significantly decreased ATAC-seq signals in TKO_PP cells. The significance was statistically determined by ZOOPS scoring coupled with hypergeometric enrichment calculations without multiple test correction. The top 10 significant motifs are shown after removing redundant motifs.

**
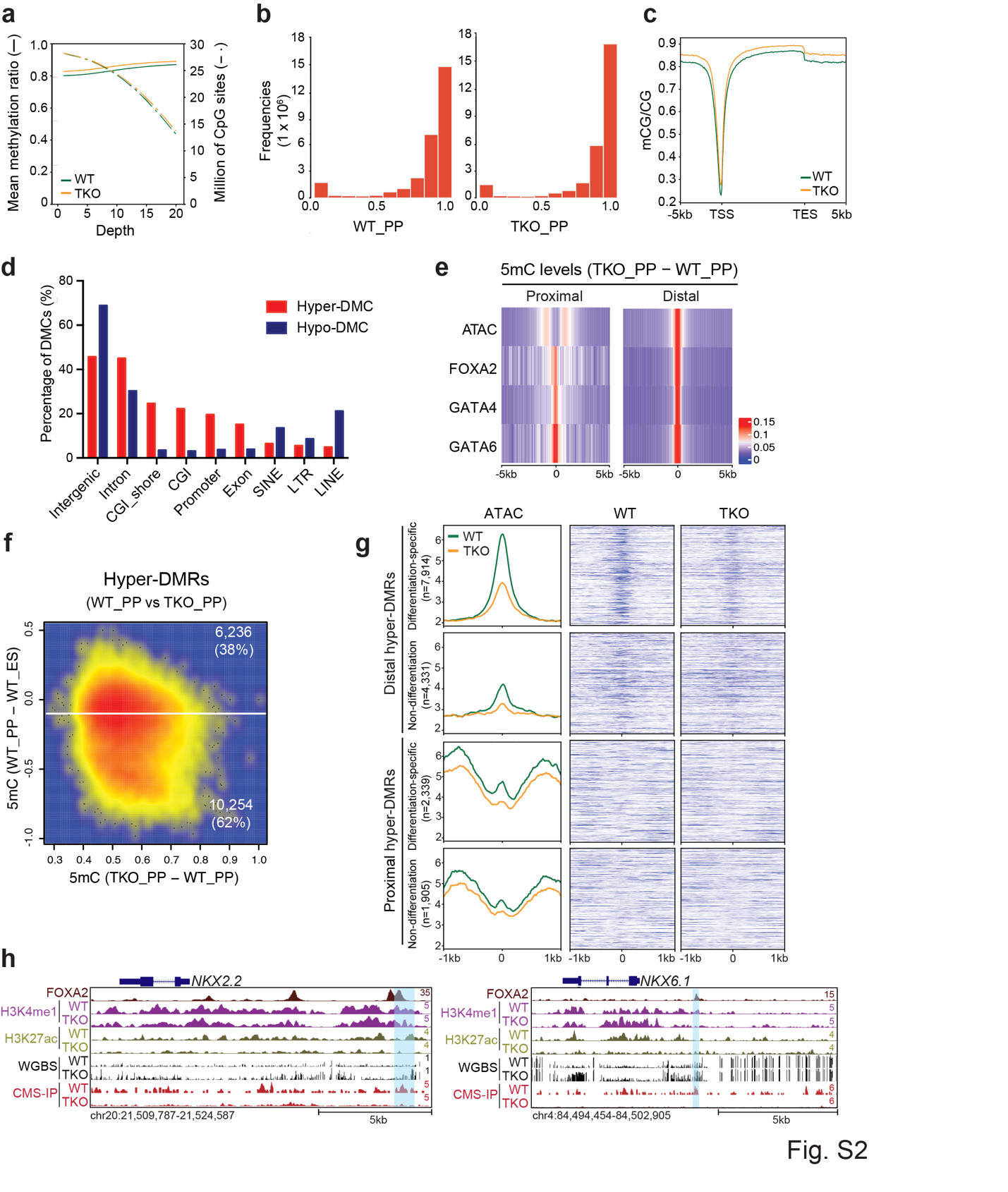
**

**Supplementary Fig. 2 Loss of TET leads to extensive hypermethylation of pancreatic regulatory elements**.

**a** CpG coverage in PP for WT and TKO cells. The x-axis represents the sequencing coverage for CpG sites. The solid line represents the average methylation ratio (5mC/C) at the corresponding coverage for each sample (left y-axis). The dashed line represents the number of CpGs over the corresponding coverage for each sample (right y-axis). **b** Bar plots showing the distribution of methylation in WT_PP and TKO_PP cells. **c** Mean distribution of the average methylation ratio (5mC/C) across gene bodies in PP for WT and TKO cells. **d** Distribution of hyper-DMCs (red) and hypo-DMCs (blue) at various genomic features. **e** Heatmap illustrating methylation difference between TKO_PP and WT_PP at centers of annotated genomic features (± 5 kb) for chromatin accessibility (ATAC) and TF binding (FOXA2, GATA4, and GATA6) at proximal (≤ 1 kb from TSS) and distal (> 1 kb from TSS) regions. **f** Comparison of global methylation at hyper-DMRs revealed that TKO_PP cells show differentiation-specific hyper-DMRs (n = 10,254) with decreased methylation in pancreatic progenitors (WT_PP) compared with hESCs (WT_ES) (methylation change < -0.1). **g** Average density plots and heatmaps of ATAC-seq reads at distal and proximal differentiation-specific or non-differentiation hyper-DMRs in PP for WT (green) and TKO (orange) cells. **h** Genome-browser views of *NKX2.2* and *NKX6.1* loci. Representative hyper-DMRs showing decreased 5hmC and H3K27ac signals are highlighted in blue.


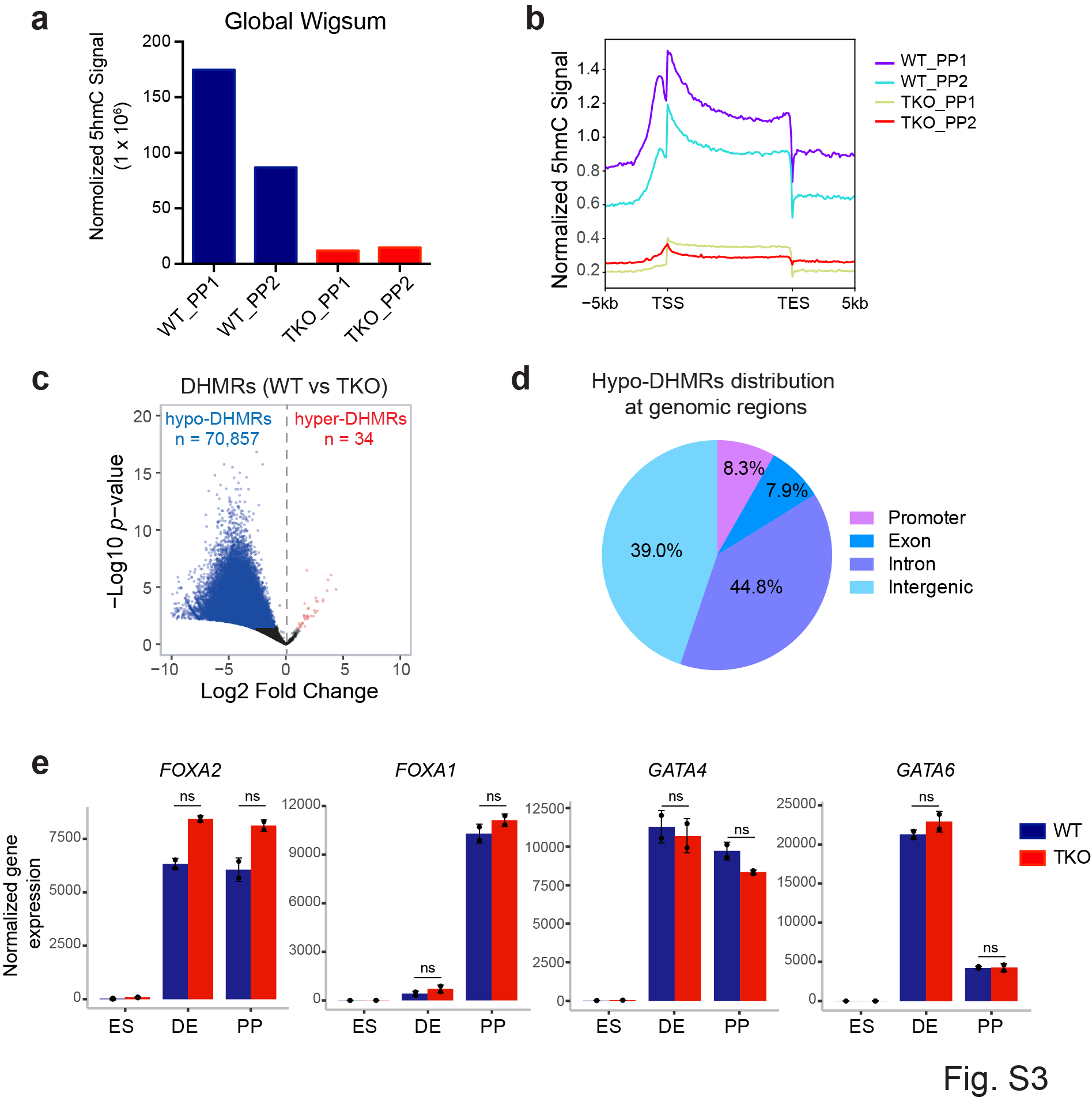


**Supplementary Fig. 3** **Hypo-hydroxymethylation at cis-regulatory elements upon TET inactivation.**

**a** Normalized 5hmC signal according to the spike-in analysis for WT_PP and TKO_PP samples with two independent replicates. **b** Distribution of 5hmC signal across gene bodies, from the transcription start site (TSS) to transcription end site (TES), in WT_PP and TKO_PP cells, with two independent replicates (± 5 kb). **c** Volcano plot of CMS-IP-seq signals illustrating differential hydroxymethylation regions (DHMRs) in TKO_PP cells relative to WT_PP cells (FDR < 0.05). **d** Diagram illustrating the overall distribution of hypo-DHMRs in promoter (within ± 1 kb from TSS), exon, intron, and intergenic regions. **e** Normalized gene expression of *FOXA1*, *FOXA2*, *GATA4*, and *GATA6* in WT and TKO cells at the ES, DE, and PP stages by RNA-seq (n = 2 replicates from independent differentiations). Statistical significance was determined using the two-sided nbinomWaldTest in the DEseq2 package, corrected for multiple testing by Benjamini–Hochberg. All bar graphs show mean ± SD.

**
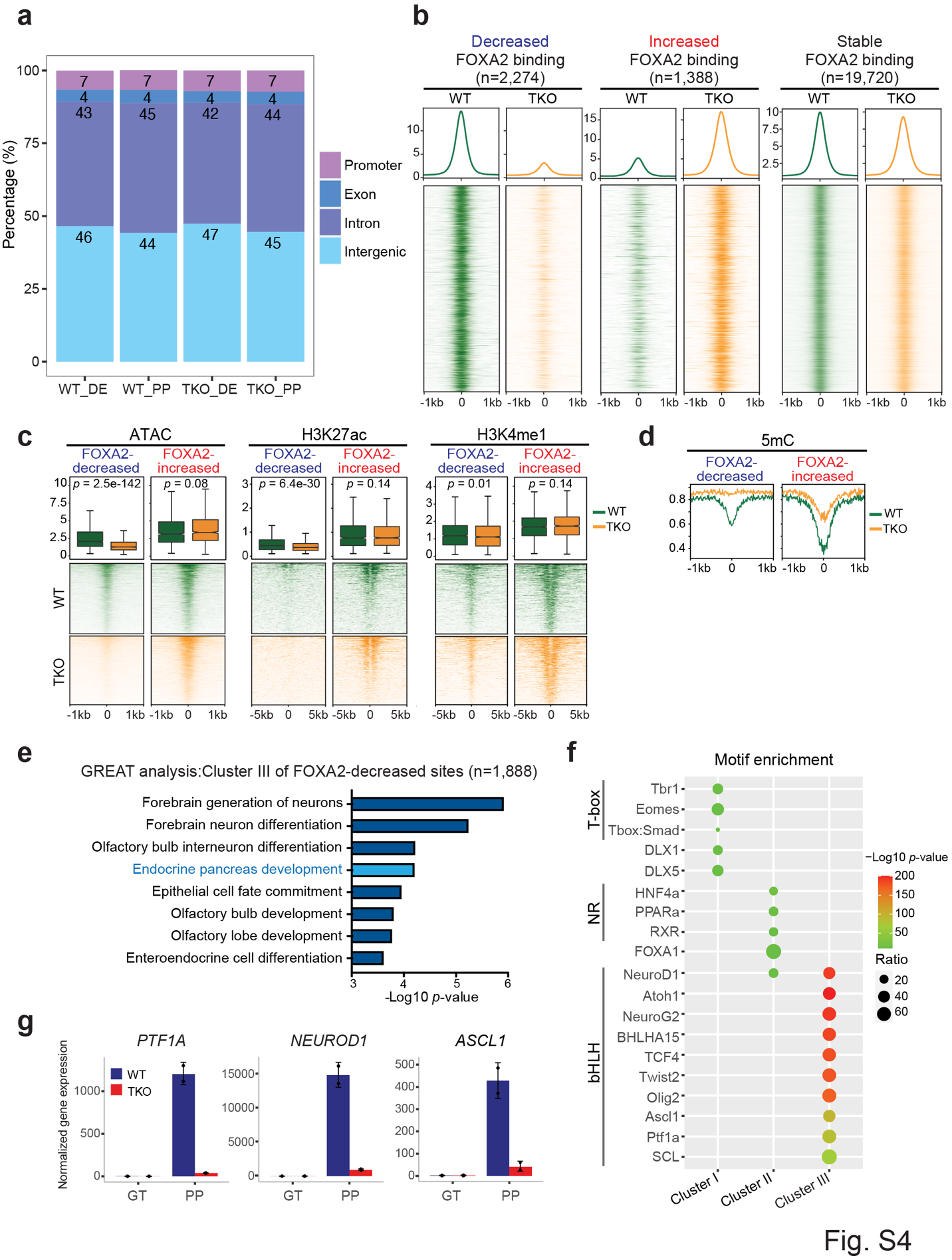
**

**Supplementary Fig. 4 De novo FOXA2 binding at pancreas-specific loci features low active chromatin modifications.**

**a ﻿**Bar graph illustrating the percentage of FOXA2 binding sites associated with different genomic features in DE and PP for WT and TKO cells. **b** Average density plots and heatmaps showing FOXA2 ChIP-seq reads in PP for WT (green) and TKO_PP (orange) cells across FOXA2-decreased, -increased, and -stable sites. **c** Box plots and heatmaps of ATAC-seq (left), H3K27ac ChIP-seq (middle), and H3K4me1 (right) reads at FOXA2-decreased (n=2,274) and -increased (n=1,388) sites in PP for WT (green) and TKO (orange) cells. Plots are centered on mean, with box encompassing 25th – 75th percentile and whiskers representing minimum to maximum range; Wilcoxon test, 2-sided (n = 2 replicates from independent differentiations). **d** ﻿Average density ﻿plot of methylation ratio (5mC/C) at FOXA2-decreased and -increased sites in PP for WT (green) and TKO (orange) cells. **e** Pathway enrichment annotations from GREAT for cluster III FOXA2-decreased sites. Benjamini-Hochberg corrected *p*-values were used. **f** Transcription factor motif enrichment analysis of genomic regions identified in cluster I, cluster II, and cluster III of FOXA2-decreased sites in TKO_PP cells. The significance was statistically determined by ZOOPS scoring coupled with hypergeometric enrichment calculations without multiple test correction. Only the top-scoring motifs are shown. The color represents *p*-values and the size of circle represents the proportion of peaks containing a TF motif in each group. **g ﻿**Normalized gene expression of *PTF1A*, *NEUROD1*, and *ASCL1* in WT and TKO cells at the GT and PP stages by RNA-seq (n = 2 replicates from independent differentiations; *p* = 1.46 ×10^-86^, *p* = 5.00 ×10^-189^, and *p* = 4.57 ×10^-19^ for *PTF1A*, *NEUROD1*, and *ASCL1* in WT compared to TKO at the PP stage, respectively; Statistical significance was determined using the two-sided nbinomWaldTest in the DEseq2 package, corrected for multiple testing by Benjamini–Hochberg). All bar graphs show mean ± SD.

**
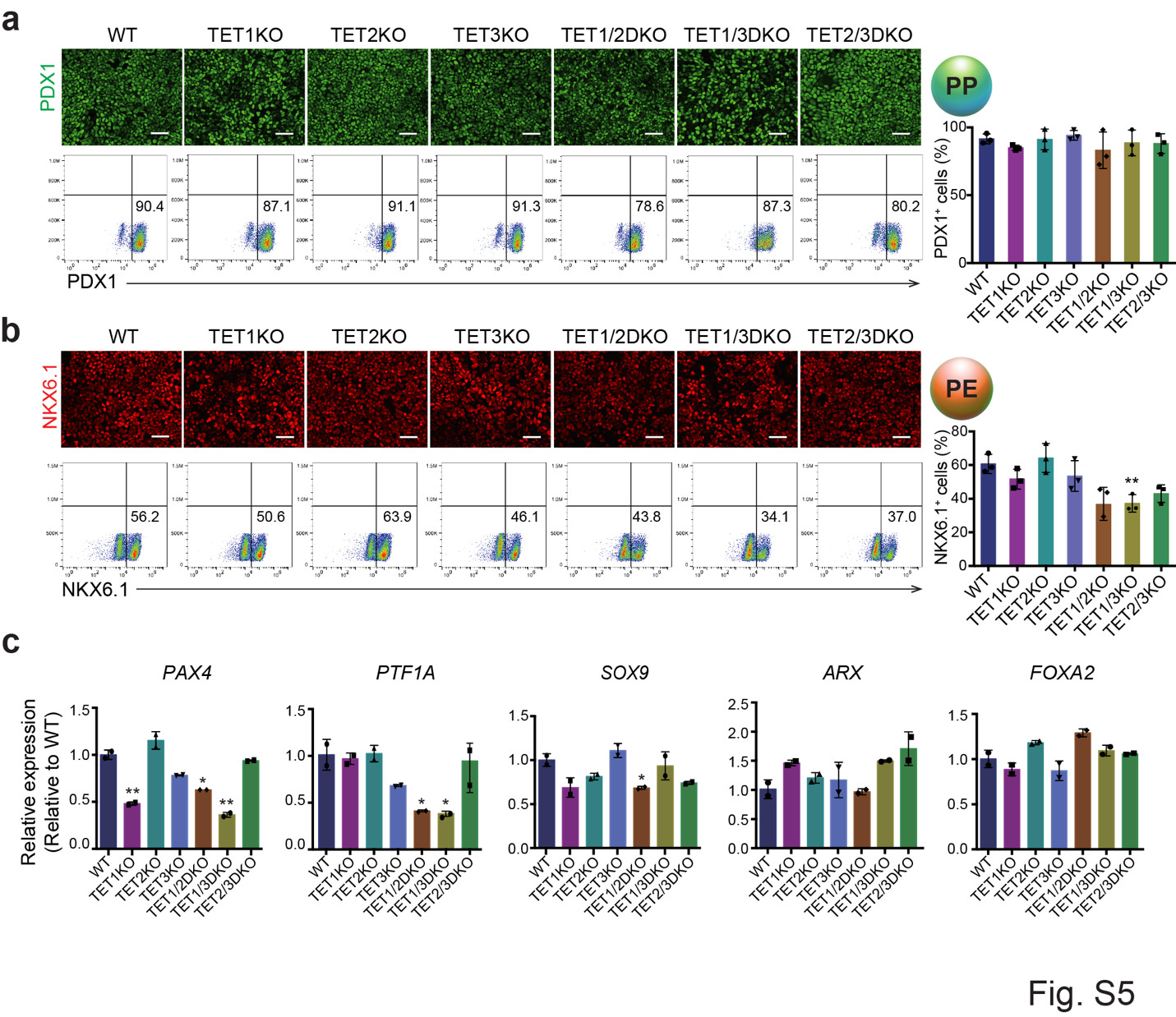
**

**Supplementary Fig. 5 TET1 is required in pancreatic β-cell specification.**

**a** Immunostaining and representative plots of flow cytometry of PDX1 at the PP stage for WT, TET1KO, TET2KO, TET3KO, TET1/2DKO, TET1/3DKO, and TET2/3DKO cells. Quantifications of the percentage of PDX1^+^ cells are shown in the right panel (n = 3 independent differentiations; scale bar = 50 μm; student’s *t*-test, 2-sided; without multiple test correction). Bar graph shows mean ± SD. **b** Immunostaining and representative plots of flow cytometry of NKX6.1 at the PE stage for WT, TET1KO, TET2KO, TET3KO, TET1/2DKO, TET1/3DKO, and TET2/3DKO cells. Quantifications of the percentage of NKX6.1^+^ cells are shown in the right panel (n = 3 independent differentiations; scale bar = 50 μm; *p* = 0.0064 for NKX6.1^+^ cells in WT compared to TET1/3DKO; student’s *t*-test, 2-sided; without multiple test correction). Bar graph shows mean ± SD. **c** Expression of *PAX4*, *PTF1A*, *SOX9*, *ARX*, and *FOXA2* in WT, TET1KO, TET2KO, TET3KO, TET1/2DKO, TET1/3DKO, and TET2/3DKO cells at the PP stage by RT-qPCR. RT-qPCR validation was performed with three independent batches of samples (*p* = 0.0072, *p* = 0.0282, and *p* = 0.0055 for *PAX4* in WT compared to TET1KO, TET1/2DKO, and TET1/3DKO, respectively; *p* = 0.0418 and *p* = 0.0408 for *PTF1A* in WT compared to TET1/2DKO and TET1/3DKO, respectively; *p* = 0.0401 for SOX9 in WT compared to TET1/2DKO; student’s *t*-test, 2-sided; without multiple test correction). All bar graphs show mean ± SD.

**
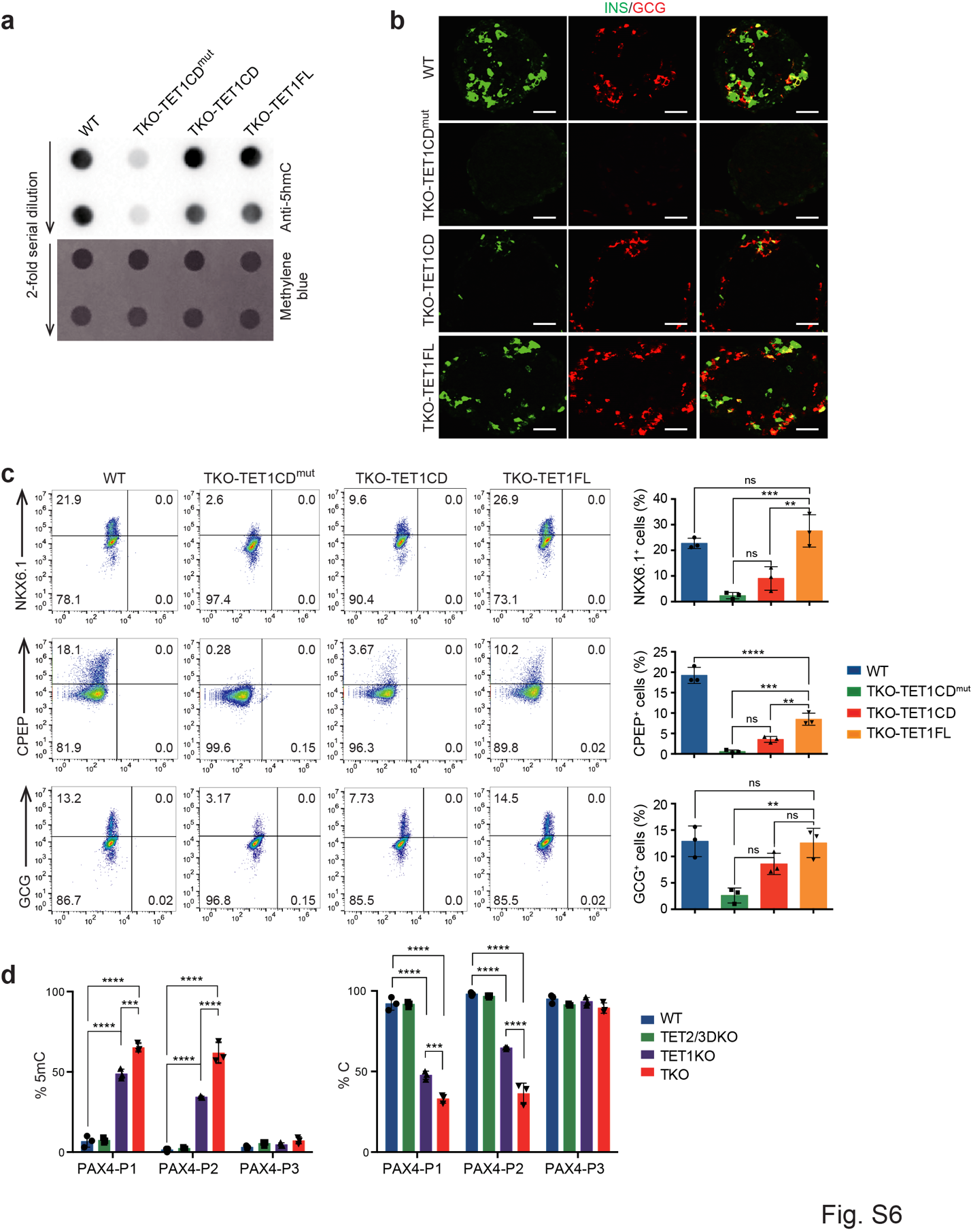
**

**Supplementary Fig. 6 Reverse hypermethylation at *PAX4* enhancer through overexpression of full-length TET1.**

**a** Analysis of global 5-hydroxymethylcytosine (5hmC) levels (top) in WT, TKO-TET1CD^mut^, TKO-TET1CD, and TKO-TET1FL cells by 5hmC dot blot analysis. The bottom panel shows methylene blue staining using the total amount of input DNA as the loading control. **b** Immunostaining of insulin (INS) and glucagon (GCG) at the PE stage for WT, TKO-TET1CD^mut^, TKO-TET1CD, and TKO-TET1FL cells (n = 3 independent differentiations; scale bar = 50 μm). **c** Representative plots of flow cytometry of NKX6.1, human C-peptide (CPEP), and glucagon (GCG) in WT, TKO-TET1CD^mut^, TKO-TET1CD, and TKO-TET1FL cells at the PE stage. Quantifications of the percentage of NKX6.1^+^, CPEP^+^, or GCG^+^ cells are shown in the right panel (n = 3 independent differentiations; *p* = 0.0003 and *p* = 0.0024 for NKX6.1^+^ cells in TKO-TET1FL compared to TKO-TET1CD^mut^ and TKO-TET1CD, respectively; *p* = 3.5 ×10^-5^, *p* = 0.0003, *p* = 0.0069 for CPEP^+^ cells in TKO-TET1FL compared to WT, TKO-TET1CD^mut^, and TKO-TET1CD, respectively; *p* = 0.0038 for GCG^+^ cells in TKO-TET1CD^mut^ compared to TKO-TET1FL; one-way ANOVA with Turkey’s multiple comparison test). All bar graphs show mean ± SD. **d** Locus-specific decreases in 5-methylcytosine (5mC) at the *PAX4* enhancer in TKO or TET1KO samples compared with TET2/3DKO samples. Percentages of unmethylated cytosine and 5mC at CCGG sites are shown (n = 3 independent differentiations; *p* = 3.4×10^-7^, *p* = 2.3×10^-8^, *p* = 0.0004, *p* = 8.5×10^-6^, *p* = 5.7×10^-8^, *p* = 3.5×10^-5^, *p* = 2.3×10^-7^, *p* = 2.3×10^-8^, *p* = 0.0010, *p* = 7.6×10^-6^, *p* = 4.7×10^-8^, and *p* = 2.6×10^-5^ for WT versus TET1KO, WT versus TKO, and TET1KO versus TKO of percentages of 5mC and unmethylated cytosine at *PAX4-P1* and *PXA4-P2* loci, respectively; one-way ANOVA with Turkey’s multiple comparison test). All bar graphs show mean ± SD.

**
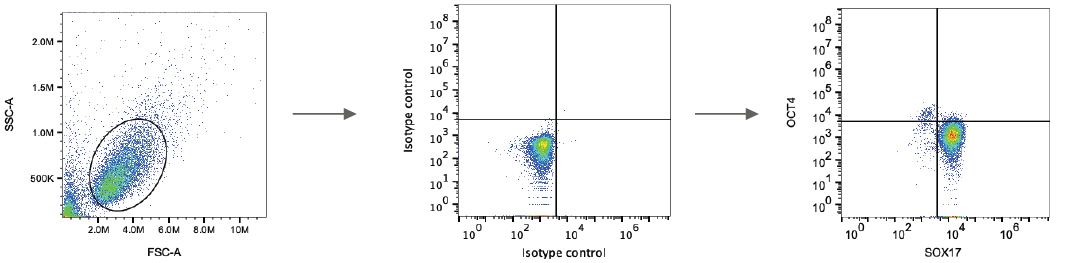
**

**Supplementary Fig. 7 Representative flow cytometry gating strategies for SOX17^+^ cells at the definitive endoderm stage.**

**Supplementary Table 1.** gRNA sequences and primers used for Sanger sequencing.

| Gene Targeted | CRISPR Target Sequence | Forward primer 5'-->3' | Reverse primer 5'-->3' |
| --- | --- | --- | --- |
| TET1 | GTGTATAGCCGGTCGGCCAT | TCATGGAGAACATCAAAAGGAA | TGCACCTGGTCACAGTGAAG |
| TET2 | TGGAGAAAGACGTAACTTCG | GGAACAGGATAGAACCAACCATGTTGAGGGC | CCCCTCCTGCTCATTCAGAATCTGAAGCTCTGG |
| TET3 | CGAAAAGGCCACCAGATCGT | ACAGGCTCAGAGCTCAGCCCAGTTGA | GGGCATTCTGGGCCATCGCAGTTGC |

**Supplementary Table 2.** Antibody sources and conditions for western blot (WB), dot blot, immunofluorescence staining (IF), co-immunoprecipitation (IP), FACS, and ChIP-seq.

| **Antibody** | **Dilution** | **Application** | **Source** | **Catalog number** |
| --- | --- | --- | --- | --- |
| OCT-4A (C30A3) | 1:1000 | IF | Cell Signaling | 2840 |
| FOXA2 | 1:1000 | IF, WB | R&D | AF2400 |
| NKX6.1 | 1:200 | IF | DSHB | F55A10 |
| PDX1 | 1:1000 | IF | Abcam | ab47383 |
| Glucagon | 1:1000 | IF | Sigma | G2654 |
| Insulin | 1:1000 | IF | Dako | A0564 |
| Somatostatin | 1:500 | IF | Abcam | ab64053 |
| OCT4-Alex647 | 1:20 | FACS | BD | 560329 |
| SOX17-PE | 1:20 | FACS | BD | 561591 |
| PDX1 | 1:200 | FACS | R&D | AF2419 |
| NKX6.1 | 1:200 | FACS | DSHB | F55A12 |
| C-peptide | 1:100 | FACS | DSHB | GN-ID4 |
| Glucagon | 1:200 | FACS | Sigma | G2654 |
| FOXA2 | 5 μg | IP | R&D | AF2400 |
| FLAG M2 | 5 μg | IP | Sigma | F1804 |
| FOXA2 | 10 μg | ChIP-seq | R&D | AF2400 |
| H3K27ac | 5 μg | ChIP-seq | Active Motif | 39133 |
| H3K4me1 | 5 μg | ChIP-seq | Abcam | ab8895 |
| 5hmC | 1:10,000 | Dot Blot | Active Motif | 39769 |
| TET1 (N3C1) | 1:1000 | WB | GeneTex | GTX124207 |

**Supplementary Table 3.** RT-qPCR primers used for mRNA expression and Epimark 5mC/C analysis.

| Genomic region | Forward primer | Reverse primer |
| --- | --- | --- |
| OCT4 | TGGGCTCGAGAAGGATGTG | GCATAGTCGCTGCTTGATCG |
| SOX2 | CCCAGCAGACTTCACATGT | CCTCCCATTTCCCTCGTTTT |
| NANOG | TGAACCTCAGCTACAAACAG | TGGTGGTAGGAAGAGTAAAG |
| SOX17 | GGCGCAGCAGAATCCAGA | CCACGACTTGCCCAGCAT |
| FOXA2 | GGGAGCGGTGAAGATGGA | TCATGTTGCTCACGGAGGAGTA |
| FOXA1 | AGGGCTGGATGGTTGTATTG | AGGCCTGAGTTCATGTTGCT |
| CXCR4 | CACCGCATCTGGAGAACCA | GCCCATTTCCTCGGTGTAGTT |
| PDX1 | CGTCCAGCTGCCTTTCCCAT | CCGTGAGATGTACTTGTTGAATAGGA |
| PTF1A | GCAGCCAGGCCCAGAAGGTC | TTCTGGGGTCCTCTGGGGTCCA |
| SOX9 | AGTACCCGCACTTGCACAAC | ACTTGTAATCCGGGTGGTCCTT |
| NKX6.1 | GCCCGCCCTGGAGGGACGCA | ACGAATAGGCCAAACGAGCCC |
| MAFB | TCGACCTGCTCAAGTTCGAC | GAGCTACACGGAGTGCTGAG |
| PAX4 | AGCAGAGGCACTGGAGAAAGAGTT | CAGCTGCATTTCCCACTTGAGCTT |
| ARX | CTGCTGAAACGCAAACAGAGGC | CTCGGTCAAGTCCAGCCTCATG |
| INS | AGCCTTTGTGAACCAACACC | GCTGGTAGAGGGAGCAGATG |
| GCG | CATTCACAGGGCACATTCAC | CGGCCAAGTTCTTCAACAAT |
| TBP | TGTGCACAGGAGCCAAGAGT | ATTTTCTTGCTGCCAGTCTGG |
| Epimark PAX4-P1 | GGGGAGGGAAAAGTGGTAAA | AGGGACAATTAGCCCCAAAC |
| Epimark PAX4-P2 | GGGGCTAATTGTCCCTCATT | GGCCTTTCTACCAGGGACTC |
| Epimark PAX4-P3 | GGTGCATTTTCGTAGGCTGT | AGAACGGTTTTGGACAGGTG |

**Supplementary Table 4.** NGS datasets used in the study.

| **Data** | **Methods** | **Sources** |
| --- | --- | --- |
| 5hmC | CMS-IP-seq | In house (WT_PP, TKO_PP), GSE97992 (ES, DE, GT, PP) |
| 5mC | WGBS | In house (WT_PP, TKO_PP), GSE80911 (H1 hESC) |
| Chromatin Accessibility | ATAC-seq | In house (WT_PP, TKO_PP) |
| Transcriptome | RNA-seq | In house (WT_ES, TKO_ES clone 2, TKO_ES clone 6) |
|  |  | In house (WT_DE, TKO_DE clone 2, TKO_DE clone 6) |
|  |  | In house (WT_GT, TKO_GT clone 2) |
|  |  | In house (WT_PP, TKO_PP clone 2, TKO_PP clone 6, TET1KO_PP) |
| H3K4me1 | H3K4me1 ChIP-seq | In house (WT_PP, TKO_PP) |
| H3K27ac | H3K27ac ChIP-seq | In house (WT_PP, TKO_PP) |
| H3K4me3 | H3K4me3 ChIP-seq | E-MTAB-1086 (PP) |
| H3K27me3 | H3K27me3 ChIP-seq | E-MTAB-1086 (PP) |
| FOXA2 | FOXA2 ChIP-seq | In house (WT_DE, TKO_DE) |
|  |  | In house (WT_GT) |
|  |  | In house (WT_PP, TKO_PP) |
| GATA4 | GATA4 ChIP-seq | GSE117136 (DE, GT, PP) |
| GATA6 | GATA6 ChIP-seq | GSE117136 (DE, GT, PP) |
| PDX1 | PDX1 ChIP-seq | GSE117136 (PP) |
| HNF6 | HNF6 ChIP-seq | GSE149148 (PP) |
