# Supplementary material for: TET1 dioxygenase is required for FOXA2-associated chromatin remodeling in pancreatic beta-cell differentiation
Source: Nat Commun. 2022 Jul 7;13:3907. doi: 10.1038/s41467-022-31611-x (PMC9263144; doi:10.1038/s41467-022-31611-x)
Supplement: Supplementary file 3 — Description of Additional Supplementary Files [file 41467_2022_31611_MOESM3_ESM.pdf]

Description of Additional Supplementary Files

Supplementary Data 1. Differentially expressed genes in TKO at PP stage.

Supplementary Data 2. Gene ontology for down-regulated genes in TKO at PP stage.

Supplementary Data 3. Differentially accessible regions in TKO at PP stage.

Supplementary Data 4. Enriched binding motifs at decreased accessible regions.

Supplementary Data 5. Hyper-DMRs in TKO (differentiation-specific and non-differentiation).

Supplementary Data 6. Hypo-DHMRs in TKO (DE-to-PP, GT(h)-to-PP, GT-to-PP(h), and PP-specific).

Supplementary Data 7. Differential FOXA2 binding in TKO compared with WT at DE stage.

Supplementary Data 8. Differential FOXA2 binding in TKO compared with WT at PP stage.

Supplementary Data 9. Enriched binding motifs at FOXA2-increased, decreased, and stable sites.

Supplementary Data 10. Differentially expressed genes in WT, TKO, and TET1KO cells at PP stage.

Supplementary Data 11. Next-Generation sequencing data statistics.
