# Supplementary material for: TET1 dioxygenase is required for FOXA2-associated chromatin remodeling in pancreatic beta-cell differentiation
Source: Nat Commun. 2022 Jul 7;13:3907. doi: 10.1038/s41467-022-31611-x (PMC9263144; doi:10.1038/s41467-022-31611-x)
Supplement: Supplementary file 15 — Reporting Summary [file 41467_2022_31611_MOESM15_ESM.pdf]

nature portfolio

Corresponding author(s): Ruiyu Xie
De-qiang Sun

Last updated by author(s): Apr 15, 2022

Reporting Summary

Nature Portfolio wishes to improve the reproducibility of the work that we publish. This form provides structure for consistency and transparency in reporting. For further information on Nature Portfolio policies, see our Editorial Policies and the Editorial Policy Checklist.

Statistics

For all statistical analyses, confirm that the following items are present in the figure legend, table legend, main text, or Methods section.

n/a	Confirmed	
☐	☒	The exact sample size (*n*) for each experimental group/condition, given as a discrete number and unit of measurement
☐	☒	A statement on whether measurements were taken from distinct samples or whether the same sample was measured repeatedly
☐	☒	The statistical test(s) used AND whether they are one- or two-sided<br>*Only common tests should be described solely by name; describe more complex techniques in the Methods section.*
☒	☐	A description of all covariates tested
☐	☒	A description of any assumptions or corrections, such as tests of normality and adjustment for multiple comparisons
☐	☒	A full description of the statistical parameters including central tendency (e.g. means) or other basic estimates (e.g. regression coefficient) AND variation (e.g. standard deviation) or associated estimates of uncertainty (e.g. confidence intervals)
☐	☒	For null hypothesis testing, the test statistic (e.g. *F*, *t*, *r*) with confidence intervals, effect sizes, degrees of freedom and *P* value noted<br>*Give P values as exact values whenever suitable.*
☒	☐	For Bayesian analysis, information on the choice of priors and Markov chain Monte Carlo settings
☒	☐	For hierarchical and complex designs, identification of the appropriate level for tests and full reporting of outcomes
☐	☒	Estimates of effect sizes (e.g. Cohen's *d*, Pearson's *r*), indicating how they were calculated

Our web collection on statistics for biologists contains articles on many of the points above.

Software and code

Policy information about availability of computer code

Data collection	CFX96 Touch Real-Time PCR Detection System (Bio-Rad): Bio-Rad CFX Manager 3.1 (v3.1.1517.0823)<br>Accuri™ C6 (BD Biosciences): Accuri™ C6 Software (v1.0.264.21)<br>Axio Observer ApoTome 2 (ZEISS): ZEN 2.3 (v2.3.69.1000)<br>ChemiDoc Imaging Systems (BioRad): Image Lab™ Touch Software (v1.2.0.12)
Data analysis	Flow cytometry data analysis: FlowJo v.10 (Tree Star, Inc), Prism 7 (GraphPad)<br>RNA-Seq data analysis: TrimGalore (v0.5.0), STAR (v2.5.3), DESeq2 (v1.28.1), ClusterProfiler (v3.16.1) package in R (v4.0.2), RSeQC (v4.0.0)<br>ATAC-Seq analysis: TrimGalore (v0.5.0), Bowtie2 (v2.4.1), Genrich (v0.5), DESeq2 (v1.28.1), BEDTools (v2.29.2), DeepTools (v2.1.0), Homer (v4.10.5), GREAT (v4.0.4)<br>CMS-IP-seq analysis: HaMiP (v0.1.1; https://github.com/lijinbio/HaMiP), bsmap (v2.89), DeepTools (v2.1.0), Homer (v4.10.5), GREAT (v4.0.4), Mmint (v0.0.1; https://github.com/lijiacd985/Mmint)<br>WGBS analysis: bsmap (v2.89), BSeQC (v1.0.3), MOABS (v1.3.8.6), DeepTools (v2.1.0), Homer (v4.10.5), GREAT (v4.0.4), Mmint (v0.0.1; https://github.com/lijiacd985/Mmint)<br>ChIP-seq analysis: TrimGalore (v0.5.0), Bowtie2 (v2.4.1), MACS2 (v2.2.7.1), BEDTools (v2.29.2), DeepTools (v2.1.0), Homer (v4.10.5), GREAT (v4.0.4), DESeq2 (v1.28.1)

For manuscripts utilizing custom algorithms or software that are central to the research but not yet described in published literature, software must be made available to editors and reviewers. We strongly encourage code deposition in a community repository (e.g. GitHub). See the Nature Portfolio guidelines for submitting code & software for further information.

Data

Policy information about availability of data

All manuscripts must include a data availability statement. This statement should provide the following information, where applicable:

- Accession codes, unique identifiers, or web links for publicly available datasets
- A description of any restrictions on data availability
- For clinical datasets or third party data, please ensure that the statement adheres to our policy

All WGBS, CMS-IP-seq, RNA-seq, ATAC-seq, and ChIP-seq datasets generated for this study have been deposited at Gene Expression Omnibus (GEO) under the accession number GSE146486 (https://www.ncbi.nlm.nih.gov/geo/query/acc.cgi?acc=GSE146486).

The following datasets used in this study were obtained from the GEO and ArrayExpress repositories – CMS-IP-Seq: DE, GT, PP (GSE97992, https://www.ncbi.nlm.nih.gov/geo/query/acc.cgi?acc=GSE97992); WGBS: H1 hESC from ENCODE project (GSE8091, https://www.ncbi.nlm.nih.gov/geo/query/acc.cgi?acc=GSE80911); ChIP-Seq: H3K4me3 and H3K27me3 in PP (E-MTAB-1086, https://www.ebi.ac.uk/arrayexpress/experiments/E-MTAB-1086/); GATA4 and GATA6 in DE, GT and PP and PDX1 in PP (GSE117136, https://www.ncbi.nlm.nih.gov/geo/query/acc.cgi?acc=GSE117136); HNF6 in PP (GSE149148, https://www.ncbi.nlm.nih.gov/geo/query/acc.cgi?acc=GSE149148).

Field-specific reporting

Please select the one below that is the best fit for your research. If you are not sure, read the appropriate sections before making your selection.

- [x] Life sciences
- [ ] Behavioural & social sciences
- [ ] Ecological, evolutionary & environmental sciences

For a reference copy of the document with all sections, see nature.com/documents/nr-reporting-summary-flat.pdf

Life sciences study design

All studies must disclose on these points even when the disclosure is negative.

Sample size	No sample size calculations were performed in this study.<br>Two biological replicates for Genome-wide experiments were determined to be sufficient based on the research community standards when the studies were performed (https://www.encodeproject.org/about/experimentguidelines).<br>The number of animals used for transplantation experiments was determined following previously study (Nair, et al 2019; PMID: 30710150). Cells from two independent differentiation experiments were collected and transplanted to a minimum of 6 mice.
Data exclusions	Only data derived from unsuccessful differentiations, as measured by immunofluorescent staining, qPCR, and Flow cytometry for the generation of endocrine cells in wild-type samples, was excluded as this data is not representative of a valid differentiation.
Replication	Three technique replicates were performed based on previous publications which had success seeing statistical significance for measure of effective similar to what we achieve with these experiment (Pasquali, et al 2014; PMID: 24413736).<br>The experiment with different TET knockout and overexpressed-lines in pancreatic differentiation of hESCs was repeated > 3 times and always resulted in the same phenotype.<br>For genome-wide sequencing data, the correlation coefficient between two biological replicates was calculated: Pearson correlations = 0.81 – 0.99 for ChIP-seq; =0.89 - 0.92 for ATAC-seq; = 0.58 - 0.61 for CMS-IP-seq, and Spearman correlation > 0.9 for RNA-seq.
Randomization	Randomization was not necessary because cells in each experiment were assayed at specific differentiation time points or in the context of specific genetic modifications.
Blinding	Blinding was not performed because values derived from all experiments were quantitative and did not require subjective interpretation.

Reporting for specific materials, systems and methods

We require information from authors about some types of materials, experimental systems and methods used in many studies. Here, indicate whether each material, system or method listed is relevant to your study. If you are not sure if a list item applies to your research, read the appropriate section before selecting a response.

Materials & experimental systems

n/a	Involved in the study
☐	☒ Antibodies
☐	☒ Eukaryotic cell lines
☒	☐ Palaeontology and archaeology
☐	☒ Animals and other organisms
☒	☐ Human research participants
☒	☐ Clinical data
☒	☐ Dual use research of concern

Methods

n/a	Involved in the study
☐	☒ ChIP-seq
☐	☒ Flow cytometry
☒	☐ MRI-based neuroimaging

Antibodies

Antibodies used

Immmunofluorescence staining:
rabbit anti-OCT4 (C30A3) Cell Signaling Cat# 2840S, RRID:AB_2167691
goat anti-FOXA2 R&D Cat# AF2400, RRID:AB_2294104
mouse anti-NKX6.1 Developmental Studies Hybridoma Bank (DSHB) Cat# F55A10, RRID:AB_532378
goat anti-PDX1 Abcam Cat# ab47383, RRID:AB_2162359
mouse anti-glucagon (GCG) Sigma Cat# G2654, RRID:AB_259852
guinea pig anti-insulin (INS) Dako Cat# A0564, RRID:AB_10013624
rabbit anti-somatostatin (SST) Abcam Cat# ab64053, RRID:AB_1143012

Flow Cytometry:
mouse anti-Oct3/4-Alexa Fluor® 647 BD Biosciences Cat# 560329, RRID:AB_1645318
mouse anti-SOX17-PE BD Biosciences Cat# 561591, RRID:AB_2688022
goat anti-PDX1 R&D Cat# AF2419, RRID:AB_355257
mouse anti-NKX6.1 Developmental Studies Hybridoma Bank (DSHB) Cat# F55A12, RRID:AB_532379
rat anti-C-peptide Developmental Studies Hybridoma Bank (DSHB) Cat# GN-ID4, RRID:AB_2255626
mouse anti-glucagon (GCG) Sigma Cat# G2654, RRID:AB_259852

ChIP-seq:
rabbit anti-H3K27ac Active Motif Cat# 39133, RRID:AB_2561016
rabbit anti-H3K4me1 Abcam Cat# ab8895, RRID:AB_306847
goat anti-FOXA2 R&D Cat# AF2400, RRID:AB_2294104

Immunoprecipitation:
goat anti-FOXA2 R&D Cat# AF2400, RRID:AB_2294104
mouse anti-FLAG M2 Sigma Cat#F1804, RRID:AB_262044

Western Blot:
goat anti-FOXA2 R&D Cat# AF2400, RRID:AB_2294104
rabbit anti-TET1(N3C1) GeneTex Cat# GTX124207, RRID:AB_11176491

Dot Blot:
rabbit anti-5hmC Active Motif Cat# 39769, RRID:AB_10013602

Validation

ChIP-seq:
rabbit anti-H3K27ac Active Motif Cat# 39133, RRID:AB_2561016
The manufacture's website shows validation that the antibody is specific for human H3K27ac protein. The antibody is highly cited for ChIP according to citeab.com and was validated by ENCODE.

rabbit anti-H3K4me1 Abcam Cat# ab8895, RRID:AB_306847
The manufacture's website shows validation that the antibody is specific for human H3K4me1 protein. The antibody is highly cited for ChIP according to citeab.com and was validated by ENCODE. Also recently rigorously evaluated for target specificity in PMID: 30244833.

goat anti-FOXA2 R&D Cat# AF2400, RRID:AB_2294104
The manufacture's website shows validation that the antibody is specific for human FOXA2 protein and cites several studies in which the antibody was used for ChIP.

Flow Cytometry:
mouse anti-Oct3/4-Alexa Fluor® 647 BD Biosciences Cat# 560329, RRID:AB_1645318
The manufacture's website shows validation that the antibody is specific for human Oct3/4 protein and cites several studies in which the antibody was used for flow cytometry.

mouse anti-SOX17-PE BD Biosciences Cat# 561591, RRID:AB_2688022
The manufacture's website shows validation that the antibody is specific for human SOX17 protein and cites several studies in which the antibody was used for flow cytometry.

goat anti-PDX1 R&D Cat# AF2419, RRID:AB_355257
The manufacture's website shows validation that the antibody is specific for human PDX1 protein and cites several studies in which the antibody was used for flow cytometry.

mouse anti-NKX6.1 Developmental Studies Hybridoma Bank (DSHB) Cat# F55A12, RRID:AB_532379
The manufacture's website shows validation that the antibody is specific for human NKX 6.1 protein and cites several studies in which the antibody was used for flow cytometry.

mouse anti-glucagon (GCG) Sigma Cat# G2654, RRID:AB_259852
The manufacture's website shows validation that the antibody is specific for human GCG protein and cites several studies in which the antibody was used for flow cytometry.

rat anti-C-peptide Developmental Studies Hybridoma Bank (DSHB) Cat# GN-ID4, RRID:AB_2255626
The manufacture's website shows validation that the antibody is specific for human C-peptide protein and cites several studies in which the antibody was used for flow cytometry.

Immunofluorescence (IF) staining:
rabbit anti-somatostatin (SST) Abcam Cat# ab64053, RRID:AB_1143012
This antibody was validated in-house for IF using human samples (PMID:29272356; PMID: 32094658). The antibody is highly specific for endocrine cells in the pancreatic islet. The manufacture's website shows validation that the antibody is specific for the human SST protein. The antibody is highly cited for IF in human tissue according to citeab.com.

guinea pig anti-insulin (INS) Dako Cat# A0564, RRID:AB_10013624
This antibody was validated in-house for IF using human tissue (PMID: 21829703; PMID: 23318056). The antibody is highly specific for endocrine cells in the pancreatic islet. The manufacture's website shows validation that the antibody is specific for human INS protein. The antibody is highly cited for IF according to citeab.com.

mouse anti-glucagon (GCG) Sigma Cat# G2654, RRID:AB_259852
This antibody was validated in-house for IF using human tissue (PMID: 23318056). The antibody is highly specific for a population of endocrine cells in the pancreatic islet. The manufacture's website shows validation that the antibody is specific for human GCG protein. The antibody is highly cited for IF according to citeab.com.

goat anti-PDX1 Abcam Cat# ab47383, RRID:AB_2162359
The manufacture's website shows validation that the antibody is specific for human PDX1 protein. The antibody is highly cited for IF according to citeab.com.

mouse anti-NKX6.1 Developmental Studies Hybridoma Bank (DSHB) Cat# F55A10, RRID:AB_532378
The manufacture's website shows validation that the antibody is specific for human NKX6.1 protein. The antibody is highly cited for IF according to citeab.com.

goat anti-FOXA2 R&D Cat# AF2400, RRID:AB_2294104
The manufacture's website shows validation that the antibody is specific for human FOXA2 protein and cites several studies in which the antibody was used for IF.

rabbit anti-OCT4 (C30A3) Cell Signaling Cat# 2840, RRID: AB_2167691
The manufacture's website shows validation that the antibody is specific for human OCT4 protein. The antibody is highly cited for IF according to citeab.com.

Immunoprecipitation (IP):
goat anti-FOXA2 R&D Cat# AF2400, RRID:AB_2294104
The manufacture's website shows validation that the antibody is specific for human FOXA2 protein. The antibody has been cited widely for IP.

mouse anti-FLAG M2 Sigma Cat#F1804, RRID:AB_262044
The manufacture's website shows validation that the antibody is specific for DYKDDDDK epitope tag. The antibody is highly cited for IP according to citeab.com.

Western Blot (WB):
goat anti-FOXA2 R&D Cat# AF2400, RRID:AB_2294104
The manufacture's website shows validation that the antibody is specific for human FOXA2 protein and cites several studies in which the antibody was used for WB.

rabbit anti-TET1(N3C1) GeneTex Cat# GTX124207, RRID:AB_11176491
The manufacture's website shows validation that the antibody is specific for human TET1 protein. The antibody is highly cited for WB according to citeab.com.

Dot Blot (DB):
rabbit anti-5hmC Active Motif Cat# 39769, RRID:AB_10013602
The antibody shows specific reaction with 5-hydroxymethylcytosine. The antibody is highly cited for DB according to citeab.com.

Eukaryotic cell lines

Policy information about cell lines

Cell line source(s)	H1 hESCs (WiCell WA01), WiCell Research Institute, NIHhESC-10-0043, RRID:CVCL_9771<br>HEK293T, ATCC, Cat# CRL-3216, RRID:CVCL_0063
Authentication	H1 human embryonic stem cells (hESC) experiments were conducted on passage P59-P66 cryopreserved cells. H1 hESCs were karyotyped quarterly to ensure genomic stability. In each experiment, proper differentiation of hESCs was validated by flow cytometry analysis for stage-specific markers at the definitive endoderm and pancreatic progenitor.<br>HEK293T cells were purchased from ATCC. The company validates cell line identity through STR analysis.
Mycoplasma contamination	Cell lines were tested for mycoplasma on a quarterly basis. No positive results were found.
Commonly misidentified lines<br>(See ICLAC register)	No commonly misidentified lines were used.

Animals and other organisms

Policy information about studies involving animals; ARRIVE guidelines recommended for reporting animal research

Laboratory animals	Mouse: SCID-beige (Charles River), 6-week-old, both sexes.
Wild animals	No wild animals were used in this study。
Field-collected samples	No field collected samples were used in this study.
Ethics oversight	All animal experiments were approved by the University of Macau Animal Ethics Committee (protocol UMARE-042-2020). The animals were housed in a Specific-pathogen-free animal facility in the University of Macau.

Note that full information on the approval of the study protocol must also be provided in the manuscript.

ChIP-seq

Data deposition

☒ Confirm that both raw and final processed data have been deposited in a public database such as GEO.

☒ Confirm that you have deposited or provided access to graph files (e.g. BED files) for the called peaks.

Data access links<br>*May remain private before publication.*	To review GEO accession GSE146486:<br>Go to https://www.ncbi.nlm.nih.gov/geo/query/acc.cgi?acc=GSE146486<br>Enter token alchaoiynpqnbol into the box.
Files in database submission	RNA_WT_ES1.bw<br>RNA_WT_ES2.bw<br>RNA_TKO2_ES1.bw<br>RNA_TKO2_ES2.bw<br>RNA_TKO6_ES1.bw<br>RNA_TKO6_ES2.bw<br>RNA_WT_DE1.bw<br>RNA_WT_DE2.bw<br>RNA_TKO2_DE1.bw<br>RNA_TKO2_DE2.bw<br>RNA_TKO6_DE1.bw<br>RNA_TKO6_DE2.bw<br>RNA_WT_GT1.bw<br>RNA_WT_GT2.bw<br>RNA_TKO2_GT1.bw<br>RNA_TKO2_GT2.bw<br>RNA_WT_PP1.bw<br>RNA_WT_PP2.bw<br>RNA_TKO2_PP1.bw<br>RNA_TKO2_PP2.bw<br>RNA_TKO6_PP1.bw<br>RNA_TKO6_PP2.bw

RNA_TET1KO_PP1.bw
RNA_TET1KO_PP2.bw
ATAC_WT_PP1.bw
ATAC_WT_PP2.bw
ATAC_TKO_PP1.bw
ATAC_TKO_PP2.bw
CMSIP_WT_PP1.bw
CMSIP_WT_PP2.bw
CMSIP_TKO_PP1.bw
CMSIP_TKO_PP2.bw
CMSIP_input1.bw
CMSIP_input2.bw
WGBS_WT_PP.bw
WGBS_TKO_PP.bw
H3K4me1_WT_PP1.bw
H3K4me1_WT_PP2.bw
H3K4me1_TKO_PP1.bw
H3K4me1_TKO_PP2.bw
H3K27ac_WT_PP1.bw
H3K27ac_WT_PP2.bw
H3K27ac_TKO_PP1.bw
H3K27ac_TKO_PP2.bw
ChIP-seq_input_WT_PP1.bw
ChIP-seq_input_WT_PP2.bw
ChIP-seq_input_TKO_PP1.bw
ChIP-seq_input_TKO_PP2.bw
FOXA2_WT_DE1.bw
FOXA2_WT_DE2.bw
FOXA2_WT_GT1.bw
FOXA2_WT_GT2.bw
FOXA2_WT_PP1.bw
FOXA2_WT_PP2.bw
FOXA2_TKO_DE1.bw
FOXA2_TKO_DE2.bw
FOXA2_TKO_PP1.bw
FOXA2_TKO_PP2.bw
FOXA2_input_WT_DE.bw
FOXA2_input_WT_GT.bw
FOXA2_input_WT_PP.bw
FOXA2_input_TKO_DE.bw
FOXA2_input_TKO_PP.bw
RNA_WT_ES1_R1.fq.gz
RNA_WT_ES1_R2.fq.gz
RNA_WT_ES2_R1.fq.gz
RNA_WT_ES2_R2.fq.gz
RNA_TKO2_ES1_R1.fq.gz
RNA_TKO2_ES1_R2.fq.gz
RNA_TKO2_ES2_R1.fq.gz
RNA_TKO2_ES2_R2.fq.gz
RNA_TKO6_ES1_R1.fq.gz
RNA_TKO6_ES1_R2.fq.gz
RNA_TKO6_ES2_R1.fq.gz
RNA_TKO6_ES2_R2.fq.gz
RNA_WT_DE1_R1.fq.gz
RNA_WT_DE1_R2.fq.gz
RNA_WT_DE2_R1.fq.gz
RNA_WT_DE2_R2.fq.gz
RNA_TKO2_DE1_R1.fq.gz
RNA_TKO2_DE1_R2.fq.gz
RNA_TKO2_DE2_R1.fq.gz
RNA_TKO2_DE2_R2.fq.gz
RNA_TKO6_DE1_R1.fq.gz
RNA_TKO6_DE1_R2.fq.gz
RNA_TKO6_DE2_R1.fq.gz
RNA_TKO6_DE2_R2.fq.gz
RNA_WT_GT1_R1.fq.gz
RNA_WT_GT1_R2.fq.gz
RNA_WT_GT2_R1.fq.gz

RNA_WT_GT2_R2.fq.gz
RNA_TKO2_GT1_R1.fq.gz
RNA_TKO2_GT1_R2.fq.gz
RNA_TKO2_GT2_R1.fq.gz
RNA_TKO2_GT2_R2.fq.gz
RNA_WT_PP1_R1.fq.gz
RNA_WT_PP1_R2.fq.gz
RNA_WT_PP2_R1.fq.gz
RNA_WT_PP2_R2.fq.gz
RNA_TKO2_PP1_R1.fq.gz
RNA_TKO2_PP1_R2.fq.gz
RNA_TKO2_PP2_R1.fq.gz
RNA_TKO2_PP2_R2.fq.gz
RNA_TKO6_PP1_R1.fq.gz
RNA_TKO6_PP1_R2.fq.gz
RNA_TKO6_PP2_R1.fq.gz
RNA_TKO6_PP2_R2.fq.gz
RNA_TET1KO_PP1_R1.fq.gz
RNA_TET1KO_PP1_R2.fq.gz
RNA_TET1KO_PP2_R1.fq.gz
RNA_TET1KO_PP2_R2.fq.gz
ATAC_WT_PP1_R1.fq.gz
ATAC_WT_PP1_R2.fq.gz
ATAC_WT_PP2_R1.fq.gz
ATAC_WT_PP2_R2.fq.gz
ATAC_TKO_PP1_R1.fq.gz
ATAC_TKO_PP1_R2.fq.gz
ATAC_TKO_PP2_R1.fq.gz
ATAC_TKO_PP2_R2.fq.gz
CMSIP_WT_PP1_R1.fq.gz
CMSIP_WT_PP2_R1.fq.gz
CMSIP_TKO_PP1_R1.fq.gz
CMSIP_TKO_PP2_R1.fq.gz
CMSIP_input1_R1.fq.gz
CMSIP_input2_R1.fq.gz
WGBS_WT_PP_R1.fq.gz
WGBS_WT_PP_R2.fq.gz
WGBS_TKO_PP_R1.fq.gz
WGBS_TKO_PP_R2.fq.gz
H3K4me1_WT_PP1_R1.fq.gz
H3K4me1_WT_PP1_R2.fq.gz
H3K4me1_WT_PP2_R1.fq.gz
H3K4me1_WT_PP2_R2.fq.gz
H3K4me1_TKO_PP1_R1.fq.gz
H3K4me1_TKO_PP1_R2.fq.gz
H3K4me1_TKO_PP2_R1.fq.gz
H3K4me1_TKO_PP2_R2.fq.gz
H3K27ac_WT_PP1_R1.fq.gz
H3K27ac_WT_PP1_R2.fq.gz
H3K27ac_WT_PP2_R1.fq.gz
H3K27ac_WT_PP2_R2.fq.gz
H3K27ac_TKO_PP1_R1.fq.gz
H3K27ac_TKO_PP1_R2.fq.gz
H3K27ac_TKO_PP2_R1.fq.gz
H3K27ac_TKO_PP2_R2.fq.gz
ChIP-seq_input_WT_PP1_R1.fq.gz
ChIP-seq_input_WT_PP1_R2.fq.gz
ChIP-seq_input_WT_PP2_R1.fq.gz
ChIP-seq_input_WT_PP2_R2.fq.gz
ChIP-seq_input_TKO_PP1_R1.fq.gz
ChIP-seq_input_TKO_PP1_R2.fq.gz
ChIP-seq_input_TKO_PP2_R1.fq.gz
ChIP-seq_input_TKO_PP2_R2.fq.gz
FOXA2_WT_DE1_R1.fq.gz
FOXA2_WT_DE1_R2.fq.gz
FOXA2_WT_DE2_R1.fq.gz
FOXA2_WT_DE2_R2.fq.gz
FOXA2_WT_GT1_R1.fq.gz

FOXA2_WT_GT1_R2.fq.gz
FOXA2_WT_GT2_R1.fq.gz
FOXA2_WT_GT2_R2.fq.gz
FOXA2_WT_PP1_R1.fq.gz
FOXA2_WT_PP1_R2.fq.gz
FOXA2_WT_PP2_R1.fq.gz
FOXA2_WT_PP2_R2.fq.gz
FOXA2_TKO_DE1_R1.fq.gz
FOXA2_TKO_DE1_R2.fq.gz
FOXA2_TKO_DE2_R1.fq.gz
FOXA2_TKO_DE2_R2.fq.gz
FOXA2_TKO_PP1_R1.fq.gz
FOXA2_TKO_PP1_R2.fq.gz
FOXA2_TKO_PP2_R1.fq.gz
FOXA2_TKO_PP2_R2.fq.gz
FOXA2_input_WT_DE_R1.fq.gz
FOXA2_input_WT_DE_R2.fq.gz
FOXA2_input_WT_GT_R1.fq.gz
FOXA2_input_WT_GT_R2.fq.gz
FOXA2_input_WT_PP_R1.fq.gz
FOXA2_input_WT_PP_R2.fq.gz
FOXA2_input_TKO_DE_R1.fq.gz
FOXA2_input_TKO_DE_R2.fq.gz
FOXA2_input_TKO_PP_R1.fq.gz
FOXA2_input_TKO_PP_R2.fq.gz

Genome browser session
(e.g. UCSC)

http://genome-asia.ucsc.edu/cgi-bin/hgTracks?db=hg38&lastVirtModeType=default&lastVirtModeExtraState=&virtModeType=default&virtMode=0&nonVirtPosition=&position=chr1%3A163321122%2D163322795&hgsid=757842237_3bJC65oGscAYTkjUrOyXaa3UtEGZ

Methodology

Replicates

Two biological replicates were used for ChIP-seq data analysis. Initial biological replicates used for peak calling were correlated with later biological replicates using Pearson correlation (see Methods for correlation table).

Sequencing depth

sample: RNA_WT_ES 1, total number of reads: 28071240, uniquely mapped reads: 51744808, read length: 150X2bp, type: paired-end
sample: RNA_WT_ES 2, total number of reads: 27741786, uniquely mapped reads: 51729636, read length: 150X2bp, type: paired-end
sample: RNA_TKO2_ES 1, total number of reads: 22895468, uniquely mapped reads: 42345052, read length: 150X2bp, type: paired-end
sample: RNA_TKO2_ES 2, total number of reads: 25263520, uniquely mapped reads: 46706980, read length: 150X2bp, type: paired-end
sample: RNA_TKO6_ES 1, total number of reads: 24882813, uniquely mapped reads: 46161620, read length: 150X2bp, type: paired-end
sample: RNA_TKO6_ES 2, total number of reads: 31165317, uniquely mapped reads: 57615248, read length: 150X2bp, type: paired-end
sample: RNA_WT_DE 1, total number of reads: 28493798, uniquely mapped reads: 52866450, read length: 150X2bp, type: paired-end
sample: RNA_WT_DE 2, total number of reads: 29057624, uniquely mapped reads: 54029636, read length: 150X2bp, type: paired-end
sample: RNA_TKO2_DE 1, total number of reads: 24456674, uniquely mapped reads: 45472070, read length: 150X2bp, type: paired-end
sample: RNA_TKO2_DE 2, total number of reads: 25063207, uniquely mapped reads: 44901278, read length: 150X2bp, type: paired-end
sample: RNA_TKO6_DE 1, total number of reads: 28325838, uniquely mapped reads: 52452212, read length: 150X2bp, type: paired-end
sample: RNA_TKO6_DE 2, total number of reads: 19875471, uniquely mapped reads: 36772386, read length: 150X2bp, type: paired-end
sample: RNA_WT_GT 1, total number of reads: 31683144, uniquely mapped reads: 61973492, read length: 150X2bp, type: paired-end
sample: RNA_WT_GT 2, total number of reads: 32783373, uniquely mapped reads: 63796114, read length: 150X2bp, type: paired-end
sample: RNA_TKO2_GT 1, total number of reads: 33318190, uniquely mapped reads: 64869914, read length: 150X2bp, type: paired-end
sample: RNA_TKO2_GT 2, total number of reads: 26576930, uniquely mapped reads: 51709223, read length: 150X2bp, type: paired-end
sample: RNA_WT_PP 1, total number of reads: 27896272, uniquely mapped reads: 51574395, read length: 150X2bp, type: paired-end
sample: RNA_WT_PP 2, total number of reads: 24658248, uniquely mapped reads: 45695767, read length: 150X2bp, type: paired-end

sample: RNA_TKO2_PP 1, total number of reads: 21796800, uniquely mapped reads: 40383295, read length: 150X2bp, type: paired-end
sample: RNA_TKO2_PP 2, total number of reads: 22990394, uniquely mapped reads: 42721398, read length: 150X2bp, type: paired-end
sample: RNA_TKO6_PP 1, total number of reads: 20119663, uniquely mapped reads: 37175090, read length: 150X2bp, type: paired-end
sample: RNA_TKO6_PP 2, total number of reads: 29035763, uniquely mapped reads: 53730720, read length: 150X2bp, type: paired-end
sample: RNA_TET1KO_PP 1, total number of reads: 29495191, uniquely mapped reads: 54960197, read length: 150X2bp, type: paired-end
sample: RNA_TET1KO_PP 2, total number of reads: 26380424, uniquely mapped reads: 49118791, read length: 150X2bp, type: paired-end
sample: ATAC_WT_PP 1, total number of reads: 41239029, uniquely mapped reads: 44803937, read length: 75X2bp, type: paired-end
sample: ATAC_WT_PP 2, total number of reads: 64477239, uniquely mapped reads: 70756180, read length: 75X2bp, type: paired-end
sample: ATAC_TKO_PP 1, total number of reads: 63096661, uniquely mapped reads: 82236000, read length: 75X2bp, type: paired-end
sample: ATAC_TKO_PP 2, total number of reads: 66029219, uniquely mapped reads: 89326434, read length: 75X2bp, type: paired-end
sample: CMSIP_WT_PP 1, total number of reads: 33582595, uniquely mapped reads: 15743035, read length: 75bp, type: single-end
sample: CMSIP_WT_PP 2, total number of reads: 20468375, uniquely mapped reads: 8945390, read length: 75bp, type: single-end
sample: CMSIP_TKO_PP 1, total number of reads: 17209704, uniquely mapped reads: 5795678, read length: 75bp, type: single-end
sample: CMSIP_TKO_PP 2, total number of reads: 168029561, uniquely mapped reads: 66451262, read length: 40bp, type: single-end
sample: CMSIP_Input 1, total number of reads: 49589532, uniquely mapped reads: 22285858, read length: 75bp, type: single-end
sample: CMSIP_Input 2, total number of reads: 10653059, uniquely mapped reads: 5291780, read length: 40bp, type: single-end
sample: WGBS_WT_PP, total number of reads: 461179608, uniquely mapped reads: 794793167, read length: 100X2bp, type: paired-end
sample: WGBS_TKO_PP, total number of reads: 463114679, uniquely mapped reads: 802894698, read length: 100X2bp, type: paired-end
sample: H3K4me1 WT_PP 1 , total number of reads: 52209785, uniquely mapped reads: 88967911, read length: 150X2bp, type: paired-end
sample: H3K4me1 WT_PP 2 , total number of reads: 47935718, uniquely mapped reads: 80778831, read length: 150X2bp, type: paired-end
sample: H3K4me1 TKO_PP 1 , total number of reads: 45051233, uniquely mapped reads: 76117268, read length: 150X2bp, type: paired-end
sample: H3K4me1 TKO_PP 2, total number of reads: 47256717, uniquely mapped reads: 79811162, read length: 150X2bp, type: paired-end
sample: H3K27ac WT_PP 1, total number of reads: 50868701, uniquely mapped reads: 83550475, read length: 150X2bp, type: paired-end
sample: H3K27ac WT_PP 2, total number of reads: 46046343, uniquely mapped reads: 74265102, read length: 150X2bp, type: paired-end
sample: H3K27ac TKO_PP 1, total number of reads: 50088480, uniquely mapped reads: 80619162, read length: 150X2bp, type: paired-end
sample: H3K27ac TKO_PP 2, total number of reads: 47663593, uniquely mapped reads: 77532117, read length: 150X2bp, type: paired-end
sample: ChIP-seq_Input WT_PP 1, total number of reads: 49896734, uniquely mapped reads: 80379653, read length: 150X2bp, type: paired-end
sample: ChIP-seq_Input WT_PP 2, total number of reads: 47918054, uniquely mapped reads: 77320109, read length: 150X2bp, type: paired-end
sample: ChIP-seq_Input TKO_PP 1, total number of reads: 46612189, uniquely mapped reads: 74562526, read length: 150X2bp, type: paired-end
sample: ChIP-seq_Input TKO_PP 2, total number of reads: 51397518, uniquely mapped reads: 82658089, read length: 150X2bp, type: paired-end
sample: FOXA2 WT_DE 1, total number of reads: 18749327, uniquely mapped reads: 29828133, read length: 150X2bp, type: paired-end
sample: FOXA2 WT_DE 2, total number of reads: 24565165, uniquely mapped reads: 38705627, read length: 150X2bp, type: paired-end
sample: FOXA2 WT_GT 1, total number of reads: 23760200, uniquely mapped reads: 37814390, read length: 150X2bp, type: paired-end
sample: FOXA2 WT_GT 2, total number of reads: 18409470, uniquely mapped reads: 29489287, read length: 150X2bp, type: paired-end
sample: FOXA2 WT_PP 1, total number of reads: 23515553, uniquely mapped reads: 37478582, read length: 150X2bp, type: paired-end
sample: FOXA2 WT_PP 2, total number of reads: 20673444, uniquely mapped reads: 32625027, read length: 150X2bp, type: paired-end
sample: FOXA2 TKO_DE 1, total number of reads: 19981440, uniquely mapped reads: 32498043, read length: 150X2bp, type: paired-end
sample: FOXA2 TKO_DE 2, total number of reads: 19017454, uniquely mapped reads: 30014336, read length: 150X2bp, type: paired-end
sample: FOXA2 TKO_PP 1, total number of reads: 22804190, uniquely mapped reads: 36254733, read length: 150X2bp, type: paired-end
sample: FOXA2 TKO_PP 2, total number of reads: 19538393, uniquely mapped reads: 31101023, read length: 150X2bp, type: paired-end

	sample: Input WT_DE, total number of reads: 19838086, uniquely mapped reads: 31226187, read length: 150X2bp, type: paired-end<br>sample: Input WT_GT, total number of reads: 17009981, uniquely mapped reads: 26943794, read length: 150X2bp, type: paired-end<br>sample: Input WT_PP, total number of reads: 25021007, uniquely mapped reads: 38596734, read length: 150X2bp, type: paired-end<br>sample: Input TKO_DE, total number of reads: 20318640, uniquely mapped reads: 32683908, read length: 150X2bp, type: paired-end<br>sample: Input TKO_PP, total number of reads: 19233878, uniquely mapped reads: 29958062, read length: 150X2bp, type: paired-end
Antibodies	rabbit anti-H3K27ac Active Motif Cat# 39133, RRID:AB_2561016<br>rabbit anti-H3K4me1 Abcam Cat# ab8895, RRID:AB_306847<br>goat anti-FOXA2 R&D Cat# AF2400, RRID:AB_2294104
Peak calling parameters	TrimGalore (options: --quality 20 and --length 50) was used to remove the adaptor. Bowtie2 with the '--very-sensitive' option was used for alignment, and only uniquely mapped reads were retained. Bam2wig.py was used to transform the BAM file to normalized bigwig files for visualization (option: -t 2000000000). For FOXA2 ChIP-seq, Macs2 69 was used to call ChIP-seq enriched peak regions with default parameters for each replicate. The BEDTools intersect was used to obtain the highly confident FOXA2 bound regions in two biological replicates for each sample. Then BEDTools merge was used to merge the confident peaks from WT and TKO samples to create the consensus FOXA2 bound regions at the DE and PP stage, independently.
Data quality	fold change greater than 2 and FDR < 0.05 were used to identify differentially bound peaks.
Software	TrimGalore/0.5.0, Bowtie2, MACS2, BEDTools, DeepTools/2.1.0, Homer/4.10.5, GREAT.

Flow Cytometry

Plots

Confirm that:

☒ The axis labels state the marker and fluorochrome used (e.g. CD4-FITC).

☒ The axis scales are clearly visible. Include numbers along axes only for bottom left plot of group (a 'group' is an analysis of identical markers).

☒ All plots are contour plots with outliers or pseudocolor plots.

☒ A numerical value for number of cells or percentage (with statistics) is provided.

Methodology

Sample preparation	Cells derived from hESCs were incubated with Accutase® at room temperature to obtain a single-cell suspension. Cells were washed with ice-cold buffer compromised 0.2% BSA in PBS and fixed with 4% paraformaldehyde for 20 min at 4 °C. Fixed cells were permeabilized with 1 × BD Perm/Wash Buffer™ (BD Biosciences) and stained with primary antibodies diluted in 1 × BD Perm/Wash Buffer™ for 1 h at 4 °C. Cells were subsequently washed, stained with appropriate secondary antibodies for 1 h at 4 °C, and assessed using an Accuri C6 flow cytometer (BD Biosciences).
Instrument	Accuri C6 flow cytometer (BD Biosciences)
Software	FlowJo v.10 (Tree Star, Inc)
Cell population abundance	Data for 10,000 events in the post-sorted fraction were recorded for each sample. Percentage of positive cells was determined as falling within gated regions determined to exclude negative samples.
Gating strategy	Cells were first gated on FSC-A and SSC-A to eliminate debris and cell fragments. Isotype controls were used for gating negative regions. Each sample was split evenly and stained with isotype controls for the same fluorophores used to detect target protein.

☒ Tick this box to confirm that a figure exemplifying the gating strategy is provided in the Supplementary Information.
