# Supplementary figures and images for: TET1 dioxygenase is required for FOXA2-associated chromatin remodeling in pancreatic beta-cell differentiation
Source: Nat Commun. 2022 Jul 7;13:3907. doi: 10.1038/s41467-022-31611-x (PMC9263144; doi:10.1038/s41467-022-31611-x)

Fig. 6c

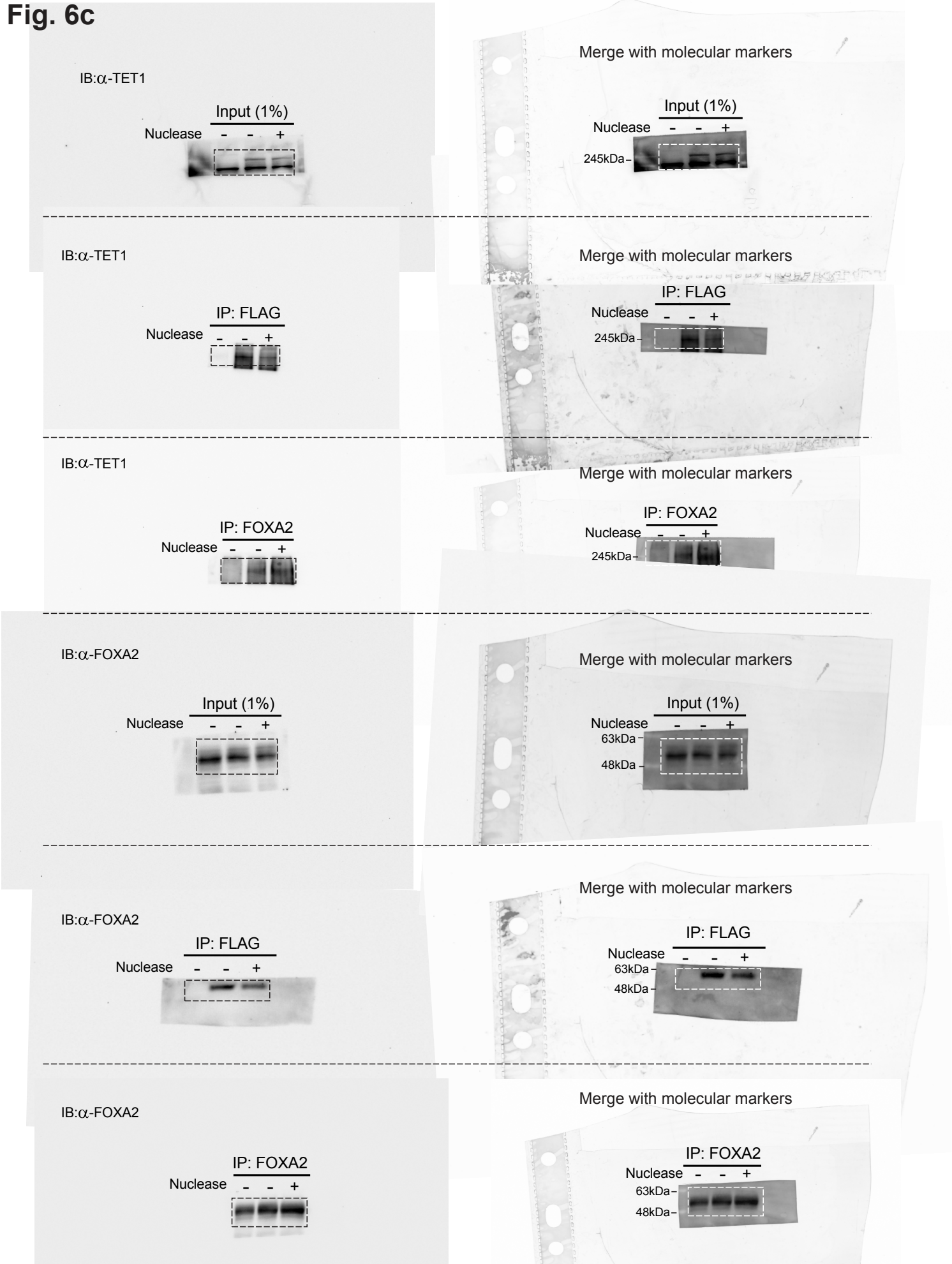

### Supplementay Fig. 1b

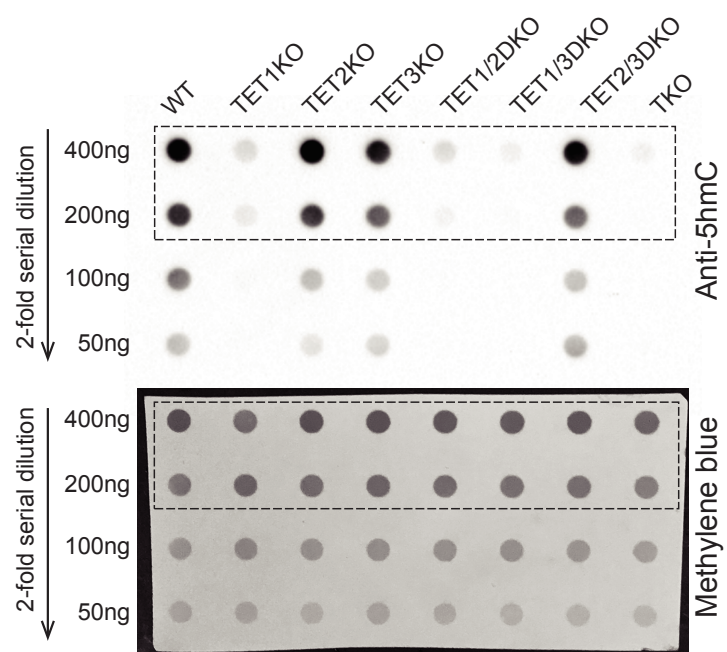

Supplementary Fig. 6a

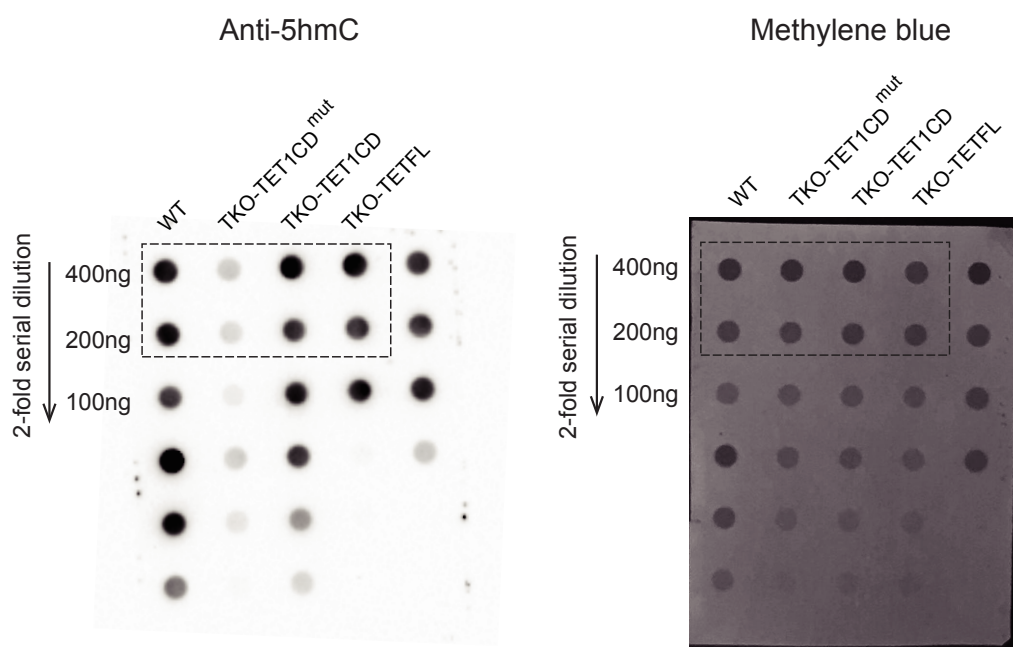

Supplement: Supplementary file 16 — Source Data [file 41467_2022_31611_MOESM16_ESM.zip › Source Data blots.pdf]
